# *Nudix hydrolase 14* influences plant development and grain chalkiness in rice

**DOI:** 10.3389/fpls.2022.1054917

**Published:** 2022-12-08

**Authors:** Yiran Liu, Wan Zhang, Youhang Wang, Liling Xie, Qiuxin Zhang, Jingjing Zhang, Weiyan Li, Meifeng Wu, Jingsong Cui, Wenyi Wang, Zemin Zhang

**Affiliations:** State Key Laboratory for Conservation and Utilization of Subtropical Agro-Bioresources, Guangdong Provincial Key Laboratory of Plant Molecular Breeding, College of Agriculture, South China Agricultural University, Guangzhou, China

**Keywords:** rice, Nudix hydrolases, grain quality, leaf angle, lignin

## Abstract

Nudix hydrolases (NUDX) can hydrolyze a wide range of organic pyrophosphates and are widely distributed in various organisms. Previous studies have shown that NUDXs are extensively involved in biotic and abiotic stress responses in different plant species; however, the role of NUDXs in plant growth and development remains largely unknown. In the present study, we identified and characterized OsNUDX14 localized in the mitochondria in rice. Results showed that *OsNUDX14* is constitutively expressed in various tissues and most strongly expressed in mature leaves. We used CRISPR/Cas9 introducing mutations that editing *OsNUDX14* and its encoding product. *OsNUDX14-*Cas9 (*nudx14*) lines presented early flowering and a larger flag leaf angle during the reproductive stage. In addition, *OsNUDX14* affected grain chalkiness in rice. Furthermore, transcript profile analysis indicated that *OsNUDX14* is associated with lignin biosynthesis in rice. Six major haplotypes were identified by six *OsNUDX14* missense mutations, including Hap_1 to Hap_6. Accessions having the Hap_5 allele were geographically located mainly in South and Southeast Asia with a low frequency in the *Xian*/*indica* subspecies. This study revealed that *OsNUDX14* is associated with plant development and grain chalkiness, providing a potential opportunity to optimize plant architecture and quality for crop breeding.

## Introduction

Nudix hydrolases (NUDX) are a superfamily of hydrolytic enzymes capable of cleaving nucleoside diphosphates linked to other moiety(x) (Bessman et al., 1996). NUDX are divided into subfamilies based on their major substrates, including adenosine diphosphate ribose (ADP-ribose), dinucleoside polyphosphates, nucleotide sugars, and deoxynucleoside triphosphates (Bessman et al., 1996; McLennan, 2006; Ogawa et al., 2008). Because nucleoside diphosphates are generally considered as toxic metabolic intermediates and signaling molecules, NUDX may act as housecleaning enzymes to cope with excessive nucleoside diphosphate and maintain cellular homeostasis (Fonseca and Dong, 2014).

Many studies have revealed the enzymatic properties and physiological roles of NUDX in various organisms, such as bacteria and yeast. However, few studies have examined the functions of NUDX in plants, most of which have been conducted in *Arabidopsis* (Ogawa et al., 2005; Olejnik and Kraszewska, 2005; Bartsch et al., 2006; Jambunathan and Mahalingam, 2006; Jambunathan et al., 2010; Njuguna et al., 2019). For example, a comprehensive analysis of nine types of cytosolic AtNUDT proteins was performed in *Arabidopsis*, results showed that the plant Nudix family evolved differently from yeast and humans (Ogawa et al., 2005). Subsequently, the same group characterized the molecular properties of several AtNUDTs and demonstrated that AtNUDTs play diverse roles in regulating a wide range of physiological processes (Ogawa et al., 2008). Several studies have revealed that AtNUDT7 is induced by abiotic stress, including drought, salinity, plant immunity and various oxidation treatments (Bartsch et al., 2006; Jambunathan and Mahalingam, 2006; Ge et al., 2007; Ishikawa et al., 2009). Besides, *AtNUDT6* encoding ADP-ribose and nicotinamide adenine dinucleotide (NADH) pyrophosphohydrolase, positively regulates the non-expresser of pathogenesis related genes-dependent salicylic acid signaling through modulation of NADH metabolism during the plant immune response (Ishikawa et al., 2010). Recently, PpNUDX8 was identified as a negative regulator of drought stress response in peaches (He et al., 2022). Overexpression of *PpNUDX8* disrupts NAD and NAD/NADH homeostasis in tobacco, and the levels of endogenous abscisic acid are also reduced (He et al., 2022). Moreover, transcriptome and quantitative reverse transcription-polymerase chain reaction (RT-qPCR) analyses showed that 10 *VvNUDX* genes might play roles in grapevine berry development (WANG et al., 2022). In addition, overexpression of *RrNUDX1* and *RwNUDX1-2* separately enhances biosynthesis of scent volatiles in petunia and sesquiterpene biosynthesis in *Rosa × wichurana* (Sun et al., 2020a; Sheng et al., 2021). Therefore, the NUDX protein plays a diversified role in physiological and biochemical processes, such as cell homeostasis, immune regulation, and oxidative stress response.

Although, there are many NUDX proteins’ function still remaining large blank. A bacterial Nudix hydrolase, identified as adenosine diphosphate sugar pyrophophatase (ASPP), that could cleave ADP-sugars such as ADP-ribose, ADP-glucose and ADP-mannose (Moreno-Bruna et al., 2001). In plants, the ADP-glucose is designated as the glucosyl donor for starch biosynthesis (Ballicora et al., 2003). AtASPP, also named AtNUDT14, was identified as a protein with the ASPP activity (Muñoz et al., 2006). Moreover, overexpressing AtNUDT14 resulting a large ADP-glucose and starch reduction in plants firstly provided the evidence of the relation between plant Nudix hydrolases and starch metabolism (Muñoz et al., 2006). Consequently, these findings also suggested the NUDX protein plays an important role in connecting ADP-glucose metabolism with starch biosynthesis.

Rice is an important cereal grain and a staple food for more than half of the world’s population. However, the role of NUDXs in rice remains unclear. The present study characterized the *OsNUDX14* which is homologous with *AtNUDT14* involved in rice development and grain chalkiness (Yoshimura and Shigeoka, 2015). We observed *OsNUDX14* highly expressed in leaves and OsNUDX14 localized to the mitochondria. Transgenic *OsNUDX14-*Cas9 (*nudx14*) lines which knock out OsNUDX14 exhibited early flowering and a larger flag leaf angle during the reproductive stage. In addition, several genes related to the lignin biosynthesis pathway were significantly downregulated in *nudx14* plants. Our findings reveal the role of *OsNUDX14* in rice plant growth and development, and its critical role in grain chalkiness.

## Materials and methods

### Plant materials and growth conditions

*Oryza sativa* L. *Geng/japonica* ‘Nipponbare’ was used as the wild type (WT) for various experiments. WT and *nudx14* plants were grown in the paddy field of South China Agricultural University (SCAU), Guangzhou (subtropical climate), China during the early (from late February to early July) and late (from middle July to late October), or in a greenhouse under a 14 h light/10 h dark cycle at 28°C. Phenotype characteristics were measured and recorded during the natural growing seasons.

### Generation of *nudx14* mutants by the CRISPR/Cas9 system

A single target site was selected for designing the single guide RNA (sgRNA) using the CRISPR-GE subroutine (Xie et al., 2017). The sgRNA cassette driven by OsU6a was inserted into the binary pYLCRISPR/Cas9 genome-targeting system by the Golden Gate ligation (Xie et al., 2017). The pYLCRISPR/Cas9 vector was then introduced into Agrobacterium strain EHA105 and transformed into NIP calli using the Agrobacterium‐mediated strategy. The genotypes of CRISPR/Cas9 plants were analyzed by PCR and direct sequencing of the amplification products. All primers used in this study are listed in **Supplementary Table S1**.

### Investigations of agronomic traits

Heading time was determined as the number of days from sowing to heading during the late season in Guangzhou for the 80 plants of the WT and *nudx14* plants. Flag leaf angle and second leaf angle were measured at the heading stage for each of the 20 WT and *nudx14* plants. Other important rice agronomic traits were recorded after seed maturity. The plant height (length from the main panicle to the ground) and stem length of every stem were also measured from 20 WT and *nudx14* plants. The main panicles were used for panicle length and primary branch number measurements. 1,000-seed weight and grain morphology were measured from fully filled and air-dried mature seeds.

### Phylogenetic analysis

To construct the phylogenetic tree, the NUDX family protein sequences in rice were retrieved target using the hmmsearch software, and the rice protein sequence database was downloaded from Ensembl Plants (Yates et al., 2022). Multiple sequence alignments were performed using the ClustalW tool. Finally, a phylogenetic tree was constructed using MEGA-X software (MEGA, Paris, France), using the best model found (WAG model) and 1,000 bootstrap replicates.

### Expression analysis by quantitative real time PCR

Total RNA was extracted from different tissues of Nipponbare at different stages using an RNA purification kit (TRIgol reagent, BEIJING DINGGUO). Total RNA (2µg) from different samples were used to synthesiz the first strand of cDNA with the usage of TransScript^®^ One-Step gDNA Removal and cDNA Synthesis SuperMix (TransGen Biotech, Beijing, China). Real-time quantitative PCR (RT-qPCR) was performed using the SYBR^®^ Green Premix Pro Taq HS qPCR Kit (#AG11701, Accurate Biology, China) in the CFX ConnectTM Real-Time System (BIO-RAD, Hercules, CA, USA) at least three plants and three technical replications.The rice ubiquitin (UBQ) was selected as an internal control for the normalization of each RNA sample. The expression levels were calculated by the method 2^−ΔΔCt^ (Schmittgen and Livak, 2001).

### Subcellular localization

To identify the subcellular localization of NUDX14, the full-length coding sequence was amplified using primers flanked by recombinant linkers. The amplified inserts were recombined into the p35S: green fluorescent protein (GFP) vector, downstream of the 35S promoter, and in-frame with a GFP tag fused to the N-terminal of NUDX14. The recombinant vector was transformed into rice protoplasts. After 16 h of incubation at 28 °C, the GFP was evaluated using confocal laser scanning microscopy (LSM 7810 DUO and LSM 7 Live, Zeiss).

### Analysis of rice grain quality

The percentage of chalky grains and degree of chalkiness were measured using SC-E0 Grain Appearant Quality analyzer (Wseen, China). Apparent amylose content (AAC) was estimated using a modified iodine colorimetric method as described previously (Tan et al., 1999). Briefly, place 20 ± 0.05 mg of rice powder and four standard samples in a 2-mL centrifuge tube. Each sample was analyzed in triplicate. 100 µl of absolute ethanol was added to each tube to wet and scatter samples. 1.8 mL of 1 mol/L NaOH solution was added, mixed, and placed in boiling water for 10 min. Next, 100 µL of the scattered liquid was transferred to a 10-mL centrifuge tube with 9 mL of double distilled water and 200 µL of 1 mol/L acetic acid was added, mixed, and placed at room temperature for 10 min. Finally, the absorbance value was measured, and the AAC of each sample was calculated using the standard curve method.

Gel consistency (GC) was estimated as previously described (Cagampang et al., 1973). Briefly, rice powder samples (100 ± 1 mg) were added to test tubes. Triplicate measurements were performed for each sample. 0.2 mL of 0.025% thymol blue was added to the test tubes and shaken slightly. 2 mL of 0.2 mol/L KOH solution was added, and the test tubes were shaken. Then each tube was covered with a glass bead and placed in boiling water for 8 min. After boiling in a water bath, the tubes were cooled to room temperature for 5 min and immediately cooled on ice for 20 min. Finally, the tubes were placed horizontally for 1 h, and the GC was measured.

Gelatinization temperature (GT) was indirectly determined *via* an alkali digestion test as previously described (Little et al., 1958). Briefly, six fully filled milled rice samples were placed in a glass dish containing 10 mL of 1.7% KOH solution. The covered dishes were incubated at 30 °C for approximately 23 h. Then the rice appearance and disintegration were visually assessed based on the grading standards of the alkali spreading value (ASV). The pasting properties were determined using the Rapid Viscosity Analyzer (RVA) (Techmaster, Newport Scientific, Warriewood, Australia). All samples were subjected to three technical replicates.

### Nucleotide diversity analysis

3K RG CoreSNP dataset in chromosome 6 downloaded from SNP-Seek was used. Nucleotide diversity of 30 kb region surrounding by *OsNUDX14* was calculated with a sliding window method of 100 bp length.

### RNA sequencing

Flag leaves of NIP and *nudx14* plants (-59bp) were harvested at the heading stage in three biological replicates. Each replicate consisted of 10 plants from each line. The samples were frozen in liquid nitrogen and stored at -80 °C. Total RNA was extracted from these samples using the TRIzol reagent (Takara, Japan). The RNA degradation and contamination were monitored on 1% agarose gels. Then, RNA purity was checked by the NanoPhotometer^®^ spectrophotometer (IMPLEN, USA). RNA concentration was measured using Qubit^®^ RNA Assay Kit in Qubit^®^2.0 Flurometer (Life Technologies, CA, USA) and RNA integrity was assessed using the RNA Nano 6000 Assay Kit of the Bioanalyzer 2100 system (Agilent Technologies, CA, USA). A total amount of 1 µg RNA per sample was used to generate sequence libraries using NEBNext^®^ UltraTM RNA Library Prep Kit for Illumina^®^ (NEB, USA) following manufacturer’s recommendations and index codes were added to attribute sequences to each sample.

The clustering of the index-coded samples was performed on a cBot Cluster Generation System using TruSeq PE Cluster Kit v3-cBot-HS (Illumia) according to the manufacturer’s instructions. After cluster generation, the library preparations were sequenced on an Illumina Hiseq platform and 125 bp/150 bp paired-end reads were generated. The original data was filtered to clean data by fastp v 0.19.3 (Chen et al., 2018). The clean reads subsequently were compared with the reference genome by HISAT v2.1.0 (Kim et al., 2019). The FPKM was then calculated using featureCounts v1.6.2 to estimate gene expression levels (Liao et al., 2014). The differential expression analysis was determined by DESeq2 v1.22.1 with FDR < 0.05 and |log2foldchange| ≥ 1 set as the threshold for significant difference expression (Love et al., 2014). KEGG enrichment analysis was performed based on the hypergeometric test with the pathway unit. Gene ontology (GO) enrichment analysis was performed using a hypergeometric test based on GO terms.

## Results

### OsNUDX14 characteristics and expression patterns

**Figure 1:**
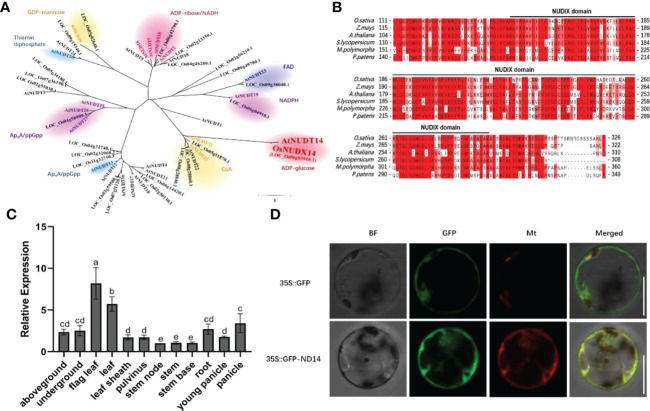
*OsNUDX14* characteristics and expression profile. **(A)** Phylogenetic tree constructed by Molecular Evolutionary Genetics Analysis 5(MEGA5) software using the neighbor-joining method. **(B)** Alignment of partial amino acid sequences among the orthologs of OsNUDIX14. The part below the black line is predicted to be a Nudix hydrolases domain using the Pfam database. **(C)** *OsNUDX14* expression in various organs was detected using quantitative reverse transcription-polymerase chain reaction (RT-qPCR). Above- and under-ground tissues represent shoots, leaves, and roots of two-week-old rice seedlings. Expression values were relative to the expression value of stem node. Data were analyzed using a two-way analysis of variance with *post hoc* Tukey tests (letters indicate significant differences between samples at p < 0.05). **(D)** OsNUDX14 subcellular localization in rice protoplast. GFP-OsNUDX14 (green channel) fluorescence was detected with mt-rkCD3-991 in red channel (Nelson et al., 2007). Bar = 10 µm.

NUDX hydrolyze a variety of organic pyrophosphates and are widely distributed across all classes of organisms. For example, AtNUDX14 can hydrolyze ADP-glucose in *Arabidopsis* (Muñoz et al., 2006). Furthermore, it regulates intracellular ADP-glucose levels linked to starch biosynthesis (Muñoz et al., 2006). A phylogenetic tree was constructed using the neighbor-joining method, and the results showed that OsNUDX14 was closely related to AtNUDX14 (**Figure 1A**). The *OsNUDX14 (Os06g0129700)* gene consists of six exons and five introns (**Figure S1**) and harbors a NUDX domain which is conserved in embryophyte (**Figure 1B**).

RT-qPCR detected temporal and spatial expression patterns of *OsNUDX14* in different tissues, including leaves, leaf sheaths, stems, roots, and panicles. The *OsNUDX14* was constitutively expressed in all tissues, and markedly high levels were observed in mature and flag leaves (**Figure 1C**), highlighting the role of *OsNUDX14* in plant growth and development, particularly in the mature leaves. AtNUDX14 was predicted to have subcellular distributions in the mitochondria; however, AtNUDX14-GFP fluorescence was colocalized with chlorophyll autofluorescence in *Arabidopsis* cells (Ogawa et al., 2008). To determine the subcellular localization of OsNUDX14, a transient expression vector harboring the GFP-OsNUDX14 fusion protein was transiently transformed into rice protoplast cells, and the fluorescence signals of GFP-OsNUDX14 were colocalized with Mito-mCherry (**Figure 1D**), indicating that OsNUDX14 was localized to the mitochondria.

### Effect of *OsNUDX14* expression on heading time

**Figure 2:**
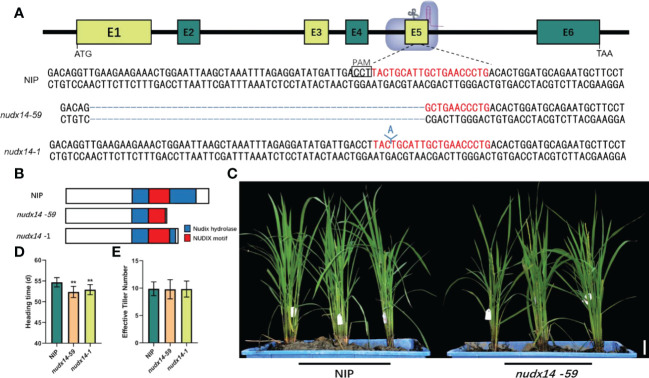
*nudx14* plants showing early flowering phenotype. **(A)** Sketch of the *OsNUDX14* and single guide RNA target sites. The protospacer adjacent motif is shown in rectangle, and different *nudx14* lines with deletions and insertions were confirmed by DNA sequencing. **(B)** 59 bp deletions (-59 bp) and 1 bp insertion (+1 bp) resulted in premature termination of OsNUDX14. **(C)** Phenotypes of wild-type (WT) and *nudx14* plants at the reproductive stage. Scar bar: 5 cm. **(D, E)** Statistical analysis of heading time **(D)** and effective tiller number **(E)** between WT and *nudx14* plants. Significance is determined by two-sided Student’s *t* test, **P < 0.01.

To investigate the NUDX14 biological function in rice, *OsNUDX14* mutant lines were generated in the *Geng/Japonica* Nipponbare (NIP) variety background using the CRISPR/Cas9 genome-editing approach, and the target site was located on the 5th exon of *OsNUDX14* (**Figure 2A**). Thus, a total of 23 transgenic plants were generated. For T0 lines, 12 out of 23 (52.17%) were identified as mutants, of which 41.67% were homozygous and the rest of lines belong to bi-allelic lines (**Table S2**). The T_2_ generations of *nudx14* lines were obtained, and deletion or insertion mutations at target sites were characterized by DNA sequencing (**Figure 2A**). Since 59 bp deletion and 1 bp insertions in *nudx14* lines resulted in premature termination of OsNUDX14 (**Figures 2A, B**), the homozygous *nudx14* line with 59 bp deletions (-59 bp) and 1 bp insertion (+1 bp) were selected for further analysis.

No significant phenotypic changes were detected between *nudx14* lines and the wild-type (WT) at the seedling and tiller stages. However, *nudx14* lines showed a considerable early flowering (nearly 4 days) phenotype compared with that of the WT at the flowering stage (**Figures 2C, D**). Other agronomic traits such as effective tiller number did not change between *nudx14* lines and WT (**Figure 2E**), indicating that OsNUDX14 may participate in regulating heading date in plants.

### *OsNUDX14* negatively modulates flag leaf angle

**Figure 3:**
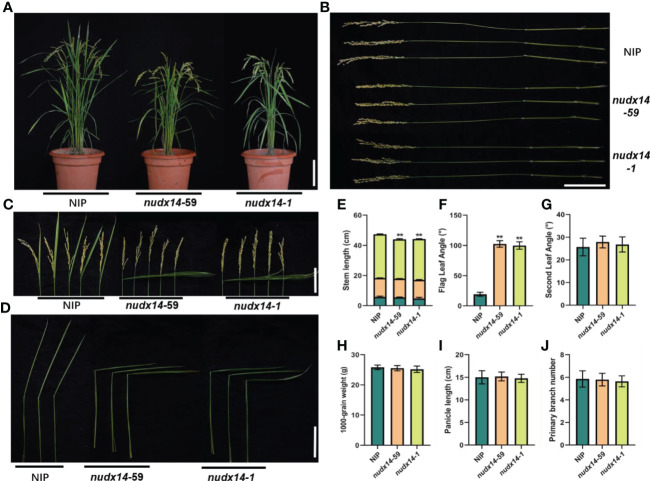
Effect of *OsNUDX14* on the regulation of plant height and flag leaf angle. **(A)** Phenotype of the wild-type (WT) and *nudx14* plants at the mature stage. Scar bar: 10 cm. **(B)** Internode length of WT and *nudx14* plants. Scar bar: 10 cm. **(C, D)** leaf angle between WT and *nudx14* plants at reproductive **(C)** and mature stage **(D)**. **(E–J)** Statistical analysis of stem length **(E)**, flag leaf angle **(F)**, second leaf angle **(G)**, 1,000-grain weight **(H)**, panicle length **(I)**, and primary branch number **(J)**. Significance is determined by two-sided Student’s *t* test, **P < 0.01.

Further characterization of the *nudx14* lines revealed substantial alterations in agronomic traits at the mature stage. *nudx14* lines had shorter plant height than that of WT and mainly because the panicle stems were truncated by 5 cm on average (**Figures 3A, B, E**). Moreover, *nudx14* plants displayed a larger flag leaf angle than that of WT (**Figures 3C, D, F**). The mean flag leaf angle of nudx14 plants is 90° while that of WT plants is only 19° (**Figures 3C, D, F**). However, no significant difference was observed in the second leaf angle (**Figure 3G**). In addition, the different plant height and leaf angle didn’t be found between the seedlings of WT plants and *nudx14* lines (**Figure S2**). Furthermore, panicle length, 1,000-grain weight, and primary branch number were not significantly different between *nudx14* lines and WT plants (**Figures 3H–J**). Overall, at the maturity stage, *nudx14* plants differed most markedly from the WT in plant height and flag leaf angle.

### *OsNUDX14* affects grain chalkiness in rice

**Figure 4:**
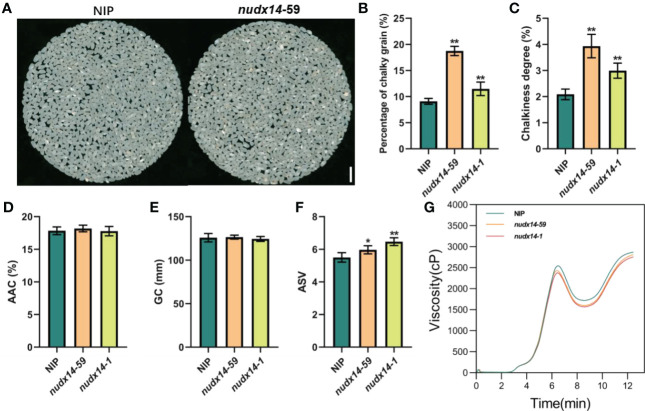
Grain quality between wild-type and *nudx14* plants. **(A)** Grain morphology in WT and *nudx14* plants. **(B–F)** Statistical analysis of percentage of chalky grain **(B)**, chalkiness degree **(C)**, apparent amylose content (ACC, **D**), gel consistency (GC, **E**) and alkali spreading value (ASV, **F**) in WT and *nudx14* plants. Significance is determined by two-sided Student’s *t* test, **P<0.01. **(G)** Viscosity (cP) measurement between WT and *nudx14* plants.

Chalkiness is a major concern in rice breeding and one of the key factors determining quality and price (Yoshioka et al., 2007). We measured the degree of chalkiness and percentage of chalky grains in *nudx14* and WT plants, results showed that both were markedly increased in *nudx14* compared to WT (**Figures 4A–C**), indicating that *OsNUDX14* is critical for grain chalkiness in rice. We further measured apparent amylose content (AAC), gel consistency (GC), alkali spreading value (ASV) and viscosity which also important for grain quality, *nudx14* plants did not differ significantly from the WT in terms of ACC and GC (**Figures 4D, E**). Additionally, ASV was increased and viscosity was subtle reduced in *nudx14* plants (**Figures 4F, G**). The increasing chalkiness, ASV and decreasing viscosity in *nudx14* plants showed that NUDX14 might regulate grain quality in rice.

### Transcript profiles reveal alterations in several metabolic pathways

**Figure 5:**
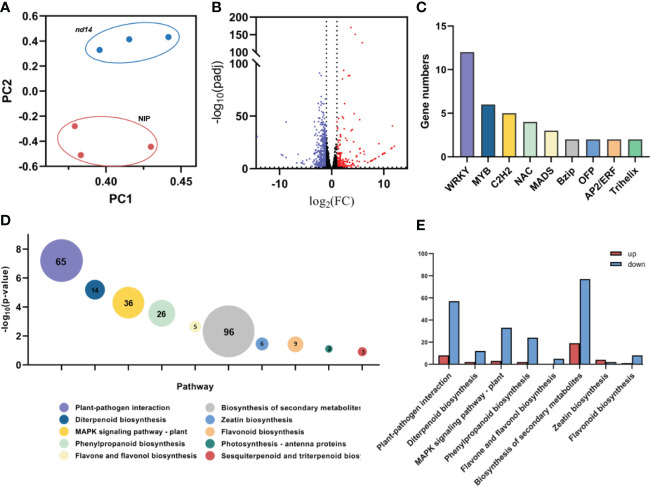
Transcriptome analysis of the wild-type and *nudx14* plants. **(A)** Principal component analysis (PCA) of transcriptomes in different samples. **(B)** Volcano plot of DEGs (259 upregulated and 759 downregulated genes) in WT and *nudx14* plants. **(C)** Number of DEGs related to transcription factors. **(D)** Top 10 pathway enrichments of DEGs analyzed by Kyoto encyclopedia of genes and genomes, bubble size and data labels indicate gene numbers. **(E)** the number of upregulated and downregulated DEGs related to top 10 pathway enrichments.

To investigate the molecular mechanism of plant growth and development controlled by *OsNUDX14* in rice, the transcriptome profiles of the WT and *nudx14* (-59 bp) leaves at the heading stage with three biological replicates were constructed, and between 44.5 and 49 million raw reads were generated. After filtering the data, between 42.5 and 46.3 million clean reads were generated, and 93.01 –93.86% of clean reads were mapped to the NIP reference genome IRGSP1.0. Furthermore, Q20 greater than 97.28% and Q30 greater than 93.05% obtained in all samples indicated reliable quality (**Figure S3**). In addition, the Pearson’s correlation coefficient test showed correlations greater than 0.99 between two groups, indicating excellent repeatability of data. A clear distinction was detected between WT and *nudx14* (-59bp) plants after principal component analysis (PCA) (**Figure 5A**).

Differentially expressed genes (DEGs) were determined using the DESeq2 package (Bioconductor, Boston, MA, USA) with the FDR < 0.05 criteria and |log2 (fold change) |≥ 1 set as thresholds. As a result, 1,018 DEGs (259 upregulated and 759 downregulated) were identified in *nudx14* (-59 bp) plants compared to those in WT (**Figure 5B**). A total of 49 transcription factors (TFs) were identified in DEGs, including 12 TFs (24.44%) were upregulated and 37 TFs (75.51%) were downregulated (**Figure 5C**). WRKY family transcription factors were mostly enriched in differentially expressed transcription factors (**Figure 5C**).

We further performed GO and KEGG enrichment analyses for the DEGs as this analysis provides an insight into the molecular understanding of how *OsNUDX14* involved in plant growth and development. KEGG analysis categorized these DEGs related to metabolic and defense pathways, such as plant-pathogen interaction, biosynthesis of secondary metabolites, MAPK signaling pathway and phenylpropanoid biosynthesis (**Figure 5D**), Notably, the number of downregulated DEGs involved in these pathways are substantial larger than upregulated DEGs (**Figure 5E**), indicating that NUDX14 might positively participates in plant growth including the biosynthesis of diverse secondary metabolites and defense. Additionally, GO enrichment analysis showed that DEGs mainly related cellular chemical homeostasis, regulation of multi-organism process, regulation of response to biotic stimulus, cell surface receptor signaling pathway, and regulation of response to external stimulus (**Figure S4**). Similarly, most of the DEGs enriched in these GO pathways being downregulated in *nudx14* plants compared to WT (**Figure S4**).

### Several genes related to lignin biosynthesis pathway displayed significant downregulation in *nudx14* plants compared to WT

**Figure 6:**
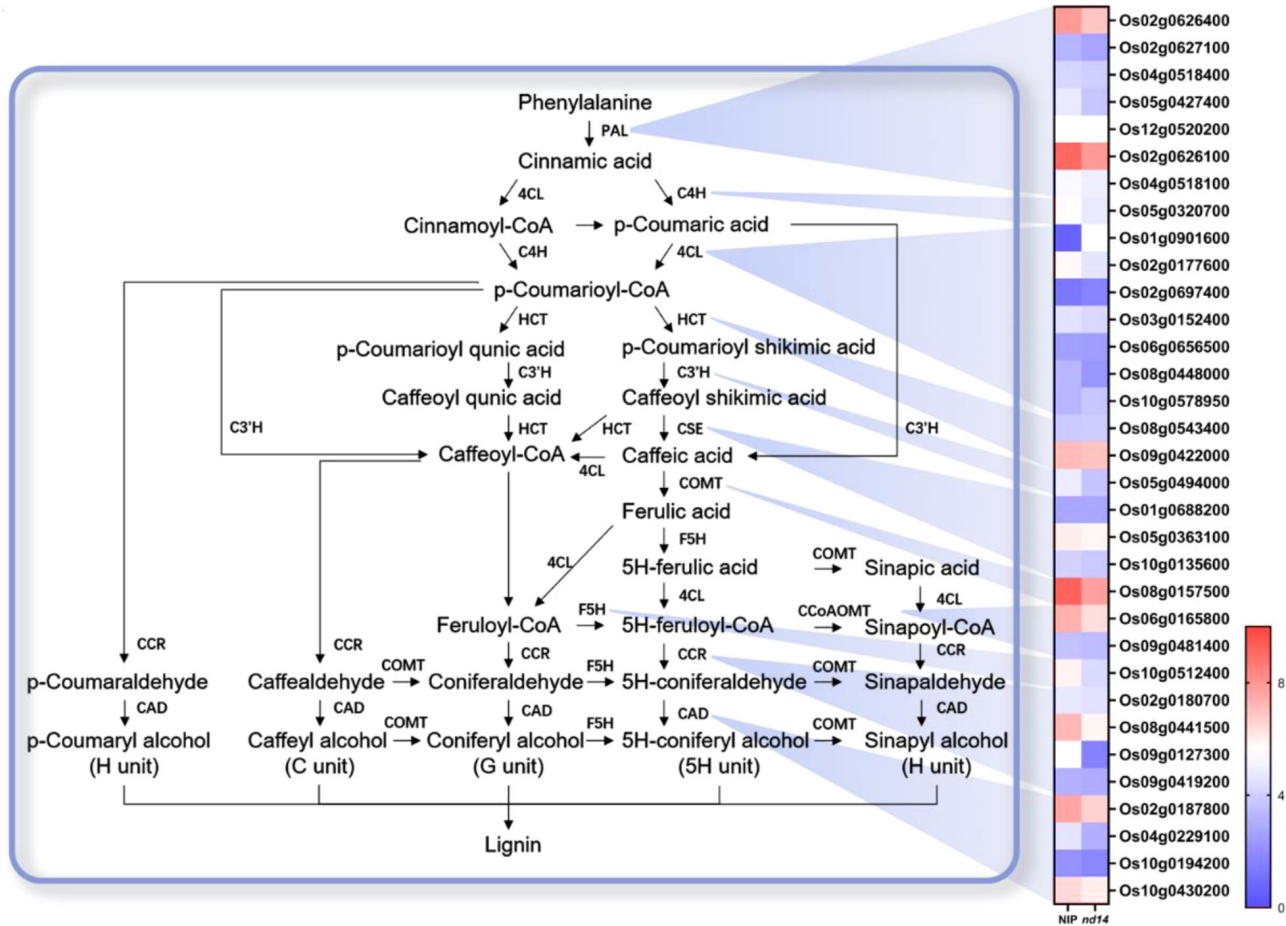
Schematic representation of gene expression in the lignin biosynthesis pathway between the wild-type (WT) and *nudx14* plants. In the heatmap for each gene, two cells represent the WT and *nudx14* plants (left to right).

As *nudx14* plants exhibited a larger flag leaf angle at the mature stage than that of WT, we investigated the transcript levels of metabolic pathways related to leaf angle regulation. Several DEGs that participate in lignin biosynthesis were identified (**Figure 6**). Lignin is a polymer of aromatic subunits thought to be synthesized from phenylalanine, converted by phenylalanine ammonia-lyase (PAL) to cinnamate. Three *PALs* were markedly downregulated in *nudx14* plants compared with those in WT plants, including *OsPAL1(Os02g0626100)*, *OsPAL2(Os02g0626400)*, and *OsPAL7(Os05g0427400)*. There were no significant differences in the majority of *Os4CL*s between the *nudx14* and WT plants, except for *Os4CL3(Os02g0177600)* downregulated in *nudx14* plants (**Figure 6**). There were no significant differences in expression levels of all *OsHCTs* and *OsCESs* between *nudx14* and WT plants (**Figure 6**). Several other key lignin biosynthetic genes, such as *OsC3H1(Os05g0494000)*, *OsCOMT (Os08g0157500)*, *F5H1(Os10g0512400)*, *OsCAD2(Os02g0187800) and OsCAD6(Os04g0229100)*, had lower transcript levels in *nudx14* plants than that in WT plants (**Figure 6**). In order to confirm the result from the transcriptome analysis, six key lignin biosynthetic genes’ relative expression levels were performed to be downregulated in both *nudx14* lines by RT-qPCR (**Figure S5**). Overall, the expression level of the lignin biosynthesis pathway was downregulated in *nudx14* plants, suggesting that *OsNUDX14* may be involved in lignin biosynthesis regulation in rice.

### A rare missense SNP might impact the leaf angle in *Xian/indica*

**Figure 7:**
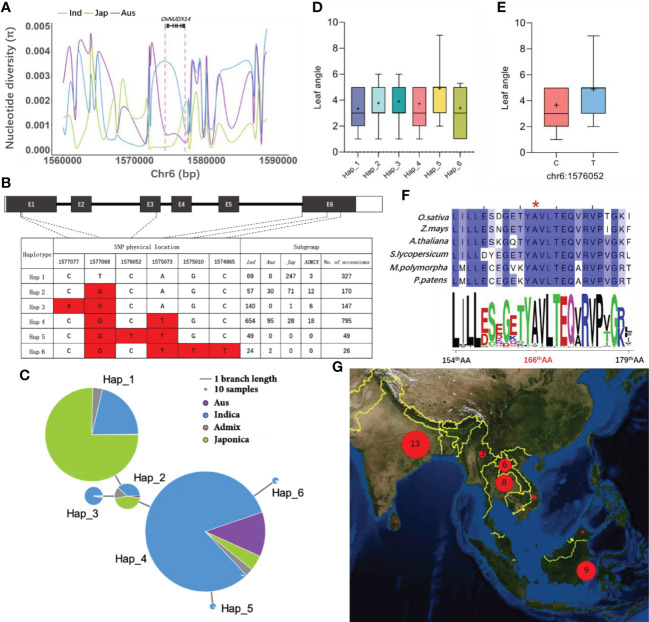
Natural variation analysis of *OsNUDX14*. **(A)** Nucleotide diversity analysis of a 30 kb region of *OsNUDX14*. Different-colored lines represent different groups; *Xian*/*Indica* (blue), *Geng*/*Japonica* (green), and *Aus* (purple) respectively. **(B)** Haplotype analysis of *OsNUDX14.* Hap_1 is the reference allele, and the red box indicates a variation as compared to Hap_1. **(C)** The haplotype network of *OsNUDX14*. The length of the branch line correlates to haplotype similarity and the size of the circle is proportional to the number of accessions. Different groups of germplasm are represented by different colors. **(D)** Leaf angle analysis in different haplotypes. The box diagram shows leaf angles with values between 10% and 90%, and the plus sign “+” indicates the mean value. **(E)** A unique missense mutation, 1576052C/T in Hap_5 displayed a larger leaf angle. **(F)** Conservation analysis of OsNUDX14’s 165^th^ AA. Multiple sequence alignment of OsNUDX14 from six different plant species and their corresponding sequence logos were calculated using 54 orthologs. The red asterisk indicates the missense amino acid variation caused by 1576052C/T. **(G)** Geographic distribution analysis of 1576052C/T. Geographic distribution of chr6:1576052-T cultivars. Red circles represent varieties and all of them are distributed in Southeast Asia and South Asia.

The most evident phenotypic change between *nudx14* lines and WT plants is the flag leaf angle. We investigated the variation in the *OsNUDX14* sequence in over 3,000 rice accessions recorded in SNP-Seek to investigate the association between *OsNUDX14* sequence variations and leaf angle phenotype. Nucleotide diversity (*Pi*) analysis of a 30 kb adjacent region of *OsNUDX14* in the core SNP data downloaded from SNP-Seek revealed that *Xian/indica* has a higher *Pi* value (0.00198) than both *Geng/japonica* (0.00086) and *aus* (0.00097) (**Figure 7A**). This suggests that the *OsNUDX14* sequence is more conserved in *Geng/japonica* and *aus* than that in *Xian/indica*. We subsequently screened the missense SNPs in the coding region of *OsNUDX14* in 1,514 different rice accessions, which have their leaf angle phenotype data recorded in SNP-Seek. Six major haplotypes were identified by six missense mutations, including Hap_1 to Hap_6 (**Figures 7B, C**). Most of Hap_3 to Hap_6 varieties were found in *indica* and Hap_5 is a rare haplotype and was found only in *indica* (**Figures 7B, C**). The varieties belonging to Hap_5 exhibited a larger leaf angle than the other haplotypes (**Figure 7D**). A unique missense mutation 1576052C/T, which results in a change in the conserved 165^th^ amino acid, from Ala to Thr in the NUDIX domain, was found only in Hap_5 (**Figure 7F**). Accessions having Hap_5 displayed a substantially larger leaf angle phenotype (**Figure 7E**; **Figure S6**). Accessions having the Hap_5 allele were geographically located mainly in Southeast Asia and South Asia with a low frequency in the *indica* subspecies (**Figure 7G**). These results indicate that 1576052C/T might be a functional rare mutation and have potential value in rice breeding.

## Discussion

NUDX are pyrophosphatases containing a highly conserved domain (Nudix box GX5Ex7REUXEEXGU), with U, a bulky hydrophobic amino acid. Evidence has shown that NUDX proteins are extensively involved in biotic and abiotic stress responses, such as drought, radiation, salinity, and pathogen attack (Fonseca and Dong, 2014). The present study identified a rice NUDIX hydrolase (NUDX14) playing a crucial role in plant growth and development.

In *Arabidopsis*, AtNUDX14 was shown to hydrolyze ADP-glucose, and its overexpression led to a substantial reduction in ADP-glucose and starch contents (Muñoz et al., 2006). In addition, AtNUDX14 exhibited pyrophosphohydrolase activity towards ADP-ribose and ADP-glucose, although its Km value was approximately 100-fold lower for ADP-ribose than that for ADP-glucose (Ogawa et al., 2008). AtNUDX14 was predicted to have subcellular distributions in the mitochondria; however, AtNUDX14-GFP fluorescence was not detected in the mitochondria and colocalized with chlorophyll autofluorescence in *Arabidopsis* cells (Ogawa et al., 2008). In the present study, OsNUDX14 was localized to the mitochondria in rice protoplast cells; however, in *Arabidopsis*, most of the NUDXs, such as AtNUDX11 and AtNUDX25, are localized in the cytosol, and very few AtNUDXs are distributed in the mitochondria (AtNUDX15), suggesting that NUDXs have diverse roles in plants (Ogawa et al., 2008).

Although the NUDIX family has been extensively studied in *Arabidopsis*, knowledge of NUDXs in other plant species remains limited. In the present study, the biological function of *OsNUDX14* was further investigated; *nudx14* lines displayed an early flowering phenotype and a larger flag leaf angle compared with those of WT during the flowering and mature stages, respectively. In addition, the knock-out of *OsNUDX14* also affects grain quality, such as ASV and viscosity (**Figure 4**), which were changed in *nudx14* plants (**Figure 4**), suggesting that *OsNUDX14* may be a key gene for rice development and grain quality. Furthermore, many studies have revealed that NUDXs play critical roles in abiotic and biotic stress responses, including heat, cold, drought, salinity, and pathogen attacks. However, the mechanism in which NUDX-mediated the regulation of plant growth and development remains unknown.

The most obvious phenotypic change between *nudx14* lines and WT is the flag leaf angle (**Figure 3**). The leaf inclination is considered an important plant architecture trait, and an appropriate leaf angle increases the efficiency of radiation capture, leading to high yields. Evidence has shown that lignin plays an extensive role in plant growth and responses to abiotic and biotic stress. Lignin is a complex phenolic heteropolymer and a key constituent of the plant’s secondary cell wall; thus, it regulates plant cell wall rigidity and hydrophobic properties, promoting mineral transport through the vascular bundle in plants (Liu et al., 2018). Reduction in lignin content may result in changes in cell wall mechanics, impairing the growth of the whole plant (Wróbel-Kwiatkowska et al., 2007). Rice SECOND-ARY WALL NAC DOMAIN PROTEIN 1 (OsSWN1) positively regulates lignin biosynthesis, and its overexpression in plants displayed an erect-leaf phenotype because of a decrease in the bending angle of the lamina joint due to lignin deposition in the leaf collar (Chai et al., 2015). Recently, rice OVATE family proteins 6 (OsOFP6) was identified as a positive regulator in modulating the expression of secondary cell wall biosynthetic genes. Its overexpression in rice led to a thinner secondary cell wall with increased lignin content (Sun et al., 2020b). *nudx14* plants displayed larger flag leaf angles than those of WT plants. In addition, lignin biosynthesis was downregulated in *nudx14* plants, suggesting that the OsNUDX14-mediated lignin biosynthesis pathway might modulate changes in leaf angle.

To reveal the underlying mechanism of the regulation of plant development by NUDX14 in rice, transcriptome profiles between *nudx14* lines and WT were analyzed. The results demonstrated that NUDX14 might regulate lignin metabolism in rice because all DEGs participating in lignin biosynthesis were downregulated, such as *OsPAL1(Os02g0626100)*, Os*4CL3(Os02g0177600), OsC3H1(Os05g0494000)*, *OsCOMT (Os08g0157500)*, *F5H1(Os10g0512400)*, *OsCAD2(Os02g0187800) and OsCAD6(Os04g0229100)*, resulting in lignin changes in the plants. For example, *4-Coumarate:coenzyme A ligase (4CL)* is a key enzyme in the phenylpropanoid metabolic pathway of monolignol biosynthesis. Os4CL3 had the fastest turnover rate in enzymatic catalysis and was the most abundantly expressed gene in all rice Os4CLs during growth (Gui et al., 2011). Suppression of *Os4CL3* expression led to significant lignin reduction and morphological changes (Gui et al., 2011). Furthermore, *Os4CL3* overexpression showed increased lignin content *(* Li et al., 2020 *)*, suggesting that it contributes to lignin biosynthesis in rice. Other DEGs, such as *F5H1* (Takeda et al., 2017) and *OsCAD2* (Yoon et al., 2017), have also been proven to be correlated with lignin levels.

Interestingly, the plant-pathogen interactions pathway was found to be the most enriched KEGG pathway (**Figure 5D**). Several lignin biosynthesis genes were reported to positively regulate disease resistance, such as *OsPAL1, OsPAL4, Os4CL3, Os4CL5* (Tonnessen et al., 2015; Zhou et al., 2018; Li et al., 2020). Downregulation of these gene expression might affect *nudx14* lines’ disease resistance. Besides, *WRKY* family of transcription factors were most enriched in DEGs (**Figure 5C**). Several differential expressed *WRKY* transcription factors were reported to regulate disease resistance such as *OsWRKY62, OsWRKY76, OsWRKY55* (Ryu et al., 2006; Peng et al., 2008; Zhang et al., 2008; Yokotani et al., 2013; Liu et al., 2016). Thus, we suspected OsNUDX14 might be associated with plant disease resistance.Poly(ADP-ribosyl)ation as a posttranslational protein modification related to the biotic stress response including lignin deposition (Adams-Phillips et al., 2010; Briggs and Bent, 2011). NUDX hydrolases with specificity for ADP-ribose can degrade degrading protein-conjugated ADP-ribose (Adams-Phillips et al., 2010; Briggs and Bent, 2011; Daniels et al., 2015; Palazzo et al., 2015). In *Arabidopsis*, AtNUDT2,6,7,10,14 have been proved to hydrolyze ADP-ribose (Ogawa et al., 2008). The subcellular localization of AtNUDT2,6,7,10 were predicted in the cytoplasm, whereas AtNUDT14 was predicted in the mitochondria (Ogawa et al., 2008). In consideration of the different localization, AtNUDT2,6,7,10 and AtNUDT14 probably function in the hydrolysis of ADP-ribose in cytoplasm and mitochondria respectively. Therefore, we suspected that the lack of OsNUDX14 influenced the NUDX hydrolases with specificity for ADP-ribose in the cytoplasm and resulted to downregulation of the genes associated with lignin biosynthesis in the cytoplasm and plant-pathogen interactions through the hydrolysis of ADP-ribose.

Our results demonstrated that NUDX14 affects grain chalkiness in rice. Many studies have shown that the formation of kernel chalkiness is controlled by multiple factors, including starch synthesis and the structure and arrangement of starch granules (Yu et al., 2015). The carbohydrate derivative catabolic process and chitin metabolic process were strongly enriched in the GO analysis (**Figure S3A**). Several genes predicted to encode glycosyl hydrolase or chitinase were all downregulated in the mutant (**Figure S3B**, **Table S3**). Therefore, the GO analysis indicates OsNUDX14 could impact the polysaccharides catabolism which might result in affecting starch metabolism. Besides, the total of 22 reported genes related to grain chalkiness and starch synthesis were found in the transcriptome profiles (**Table S4**). Only one of these genes, *OsbZIP58*, which was reported to regulate starch synthesis was markedly downregulated (Kawakatsu et al., 2009; Wang et al., 2013). However, there were half genes having slight but significant differential expression (FDR < 0.05 but |log2 (fold change)| ≤ 1), for instance *OsbZIP50* and *OsbZIP60* played important roles in the formation of grain chalkiness were slightly downregulated (Yang et al., 2022). Consequently, we suspected loss-of-function of NUDX14 might influence the expression level of genes associative with chalkiness. However, using transcriptome profiles of leaves at the heading stage could not adequately explain how NUDX14 affects grain chalkiness. Although, AtNUDX14 is capable of hydrolyzing ADP-glucose and is involved in regulating intracellular ADP-glucose levels linked to starch biosynthesis (Muñoz et al., 2006). Previous studies have shown that ADP-glucose is transported into amyloplasts to serve as the substrate for starch synthesis filling the grain with storage starch, which is an important compound in grain quality (Seng et al., 2016; Wei et al., 2017; Gann et al., 2021). Therefore, we speculated that increased grain chalkiness results from starch metabolism.

Previous studies on NUDX in plants have focused on physiological and biochemical processes such as cell homeostasis, immune regulation, and environmental stress conditions. The present study revealed NUDX14 as a crucial rice plant development and grain quality regulator. These findings may lead to a better understanding of NUDX-mediated regulation of plant characteristics, including plant height, flag leaf angle, and grain chalkiness, and optimize crop architecture for crop breeding.

## Data availability statement

The original contributions presented in the study are publicly available. This data can be found here: NCBI, PRJNA854265.

## Author contributions

YL: Data curation, investigation, formal analysis, editing. WZ: Validation and formal analysis. YW: Investigation and data curation. LX: Investigation. QZ: Investigation and data curation. JZ: Investigation and data curation. WL: Data curation. MW: Data curation. JC: Investigation and data curation. WW: Writing original draft, supervision, and data curation. ZZ: Conceptualization, resources, supervision, editing, and funding acquisition. All authors contributed to the article and approved the submitted version.
