# Supplementary material for: Nudix hydrolase 14 influences plant development and grain chalkiness in rice
Source: Front Plant Sci. 2022 Dec 8;13:1054917. doi: 10.3389/fpls.2022.1054917 (PMC9773146; doi:10.3389/fpls.2022.1054917)
Supplement: Supplementary file 1 [file DataSheet_1.docx]

Supplementary Material


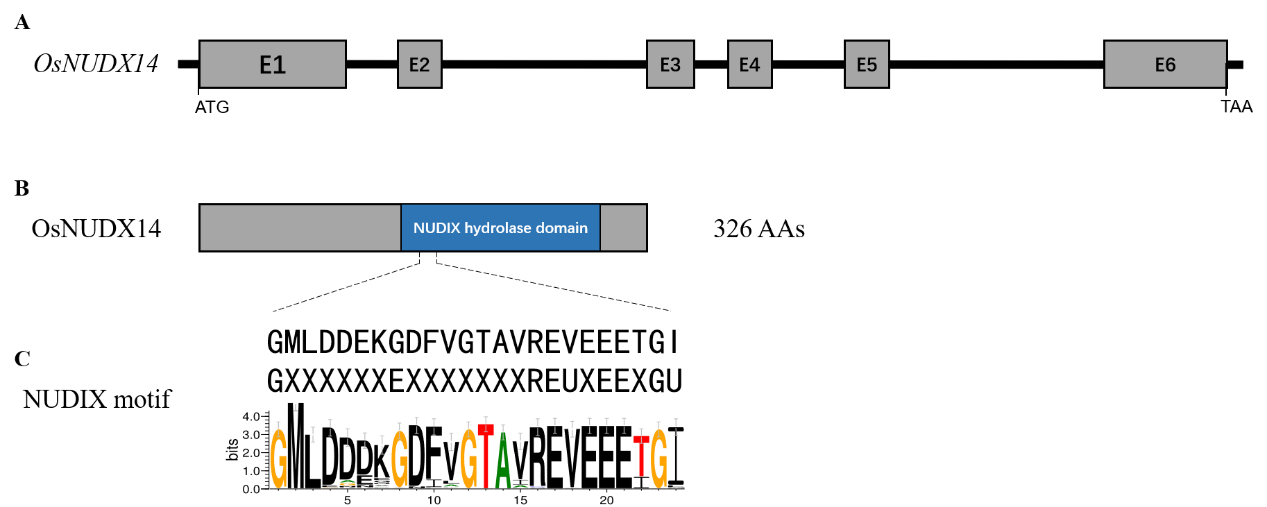


**Supplementary Figure 1****.** Gene structure of *OsNUDX14*. **(A)** Gene structure of *OsNUDX14*. **(B)** Protein structure of OsNUDX14. **(C)** The core NUDIX motif in NUDX14 orthologys’ NUDIX hydrolase domain.


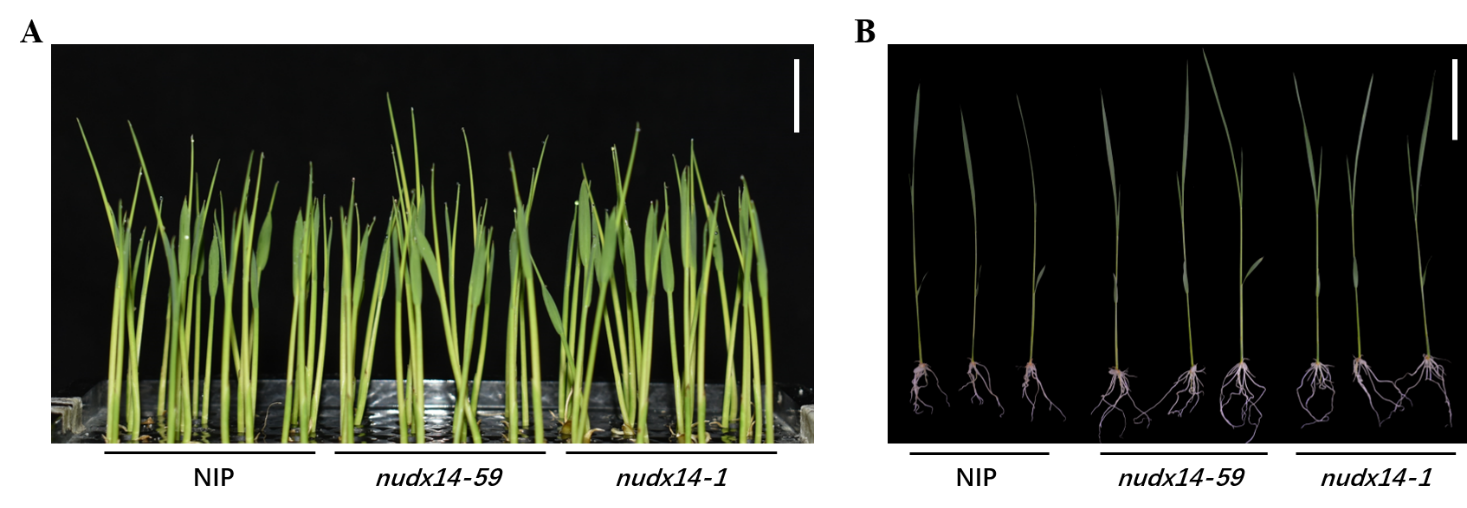


**Supplementary Figure 2.** Phenotype of NIP and *nudx* mutant lines in the two **(A)** and three **(B)** leaves periods.


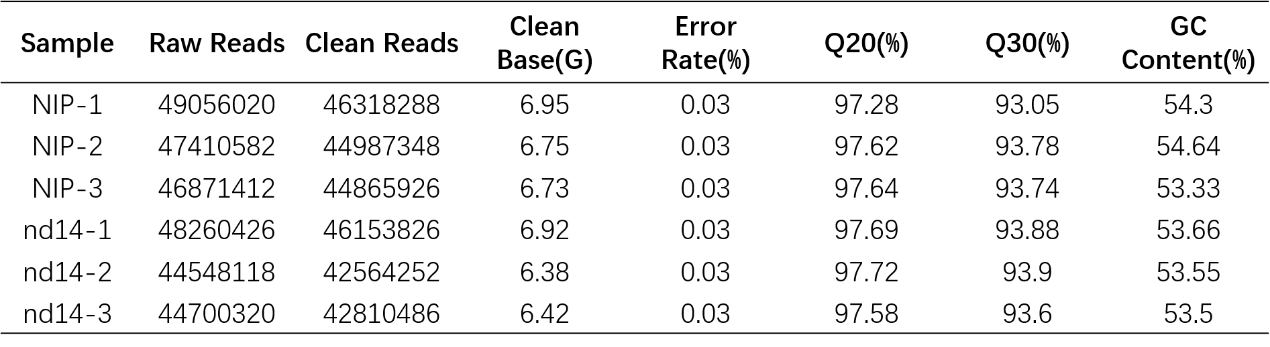


**Supplementary Figure 3.** Sequence alignment efficiency of transcript profile.


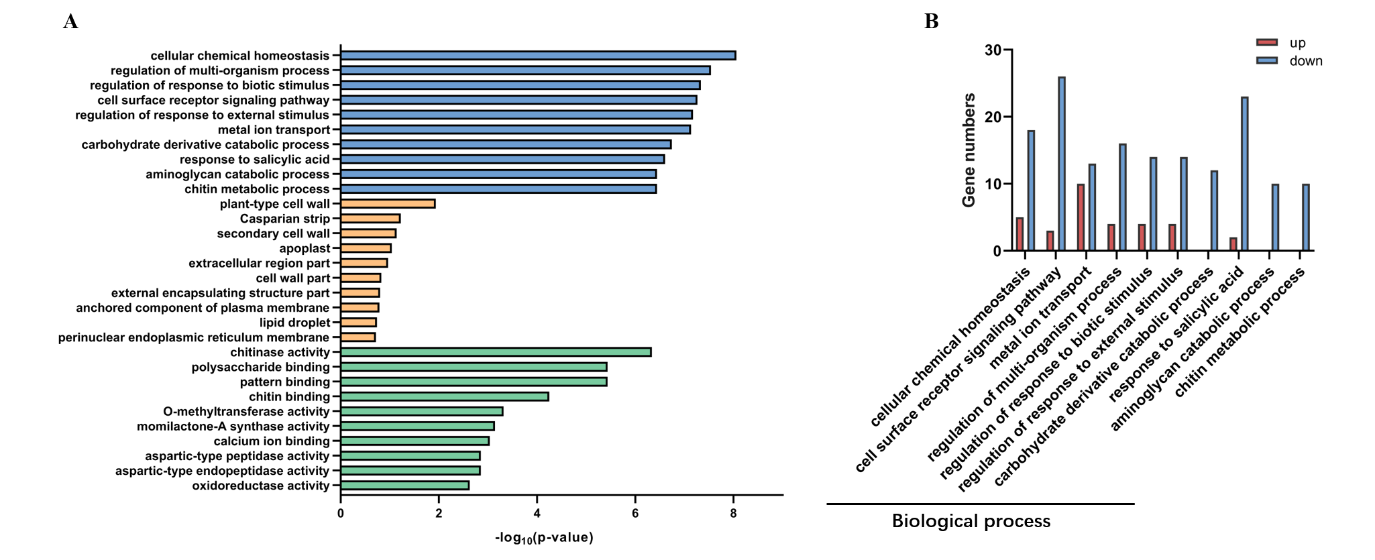


**Supplementary Figure 4.** Gene ontology (GO) analysis**. (A)** Histogram plot of gene ontology pathway enrichment analysis. Different color represents different classifications: biological process(blue), molecular function(orange) and cellular component(green). **(B)** Histogram plot of the up or down Top10 enriched pathways.

**
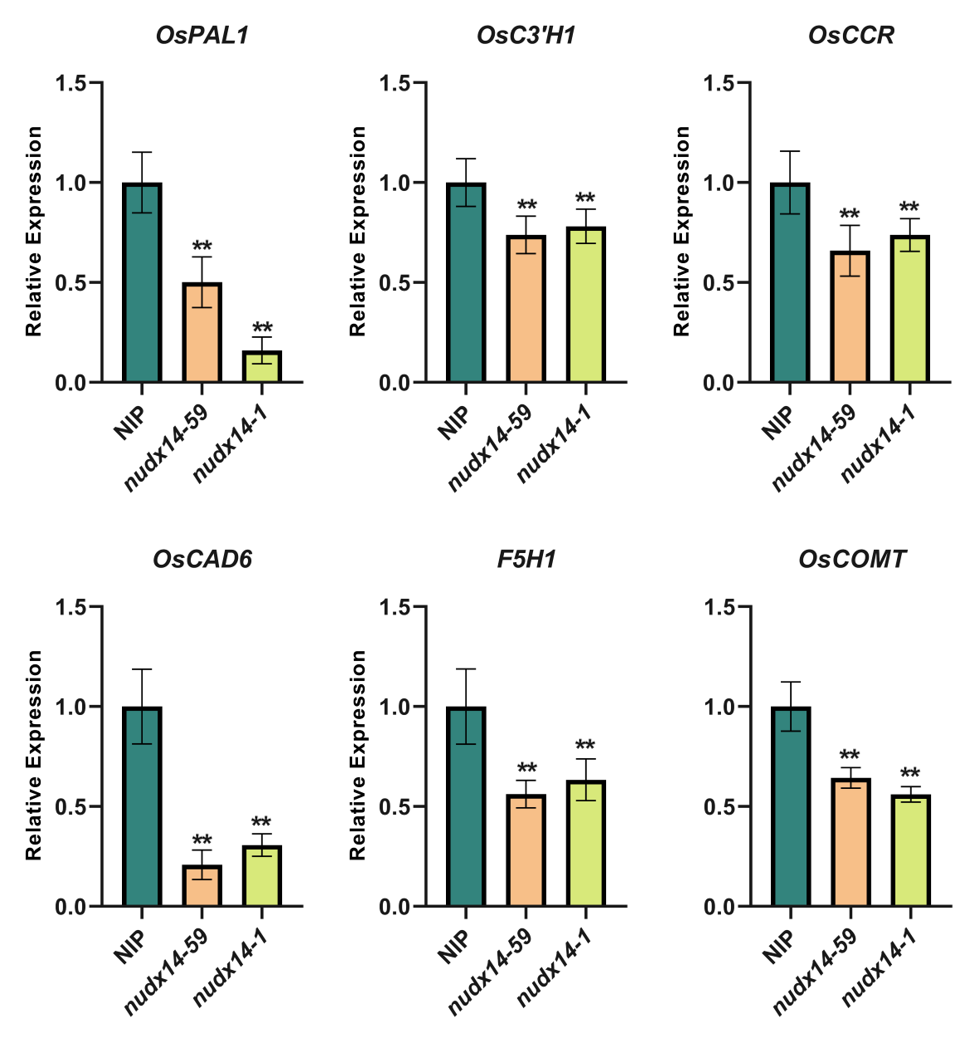
**

**Supplementary Figure 5.** The relative expression of *OsPAL1, OsC3’H1, OsCCR, OsCAD6, F5H1* and *OsCOMT* in WT and *nudx14* plants’ leaves at the reproductive stage. Significance is determined by two-sided Student’s *t* test, **P<0.01.


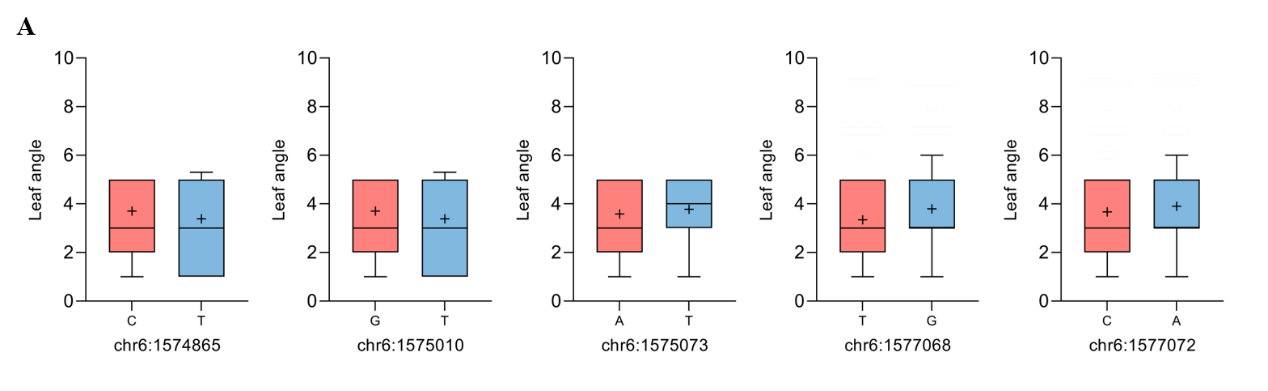


**Supplementary Figure 6.** Leaf Angle analysis distinguished by different missense mutations.

**Supplementary Table 1.** Primers used in this study.

| Purpose | Primer name | Sequence |
| --- | --- | --- |
| qRT-PCR | QPCR-NUDX14-F | ATGCAGAATGCTTCCTTCACCG |
|  | QPCR-NUDX14-R | TCCTTCCCTTGGAGAGCCCTA |
| CRISPR/Cas9 | CR-NUDX14-F | gccgCAGGGTTCAGCAATGCAGTA |
|  | CR-NUDX14-R | aaacTACTGCATTGCTGAACCCTG |
| Subcelluar localization | NGFP-NUDX14-F | CGAGCTGTACAGATCTATGGCGGCGGCGGCGG |
|  | NGFP-NUDX14-R | GGCCGCTTTAAGATCTCAAGTTGGCCGACGACGAGC |
| RT-qPCR | PAL1-qrt-F | ACATCTACGGCGTCACCACC |
|  | PAL1-qrt-R | GAGCCAGTGCCGAAGATTCC |
|  | C3H1-qrt-F | AGATCAACCACGACAACGTCC |
|  | C3H1-qrt-R | TCACCAGCTCCGCGATTCC |
|  | CCR-qrt-F | TCCGCATCCTCGCCAAGCTC |
|  | CCR-qrt-R | TCCAGCCCCAGGTCCCGGAG |
|  | CAD6-qrt-F | CTGCTCAAGGTCAACGGCAAG |
|  | CAD6-qrt-R | ATGTCCATCATCTCCTGCGTCT |
|  | F5H1-qrt-F | CGCGACAACATCAAGGCCATC |
|  | F5H1-qrt-R | TCATCTCCGCCATCGCCCAC |
|  | COMT-qrt-F | CTTCAACCGCGTCTTCAACG |
|  | COMT-qrt-R | TGGGGGAGGTCGTAGTTGAT |

**Supplementary Table 2.** Summary of mutagenesis efficiency at the target site of *OsNUDX14* in T_0_ lines.

| **No. of transgenic plants** | **No. of plants with mutation: number, ratio** | **Homozygous: number, ratio** | **Heterozygous: number, ratio** | **Bi-allelic: number, ratio** |
| --- | --- | --- | --- | --- |
| **23** | **12, 52.17%** | **5, 41.67%** | **0, 0%** | **7, 58.33%** |
